# Room temperature stable carbetocin for the prevention of postpartum haemorrhage during the third stage of labour in women delivering vaginally: study protocol for a randomized controlled trial

**DOI:** 10.1186/s13063-016-1271-y

**Published:** 2016-03-17

**Authors:** Mariana Widmer, Gilda Piaggio, Hany Abdel-Aleem, Guillermo Carroli, Yap-Seng Chong, Arri Coomarasamy, Bukola Fawole, Shivaprasad Goudar, G. Justus Hofmeyr, Pisake Lumbiganon, Kidza Mugerwa, Thi My Huong Nguyen, Zahida Qureshi, Joao Paulo Souza, A. Metin Gülmezoglu

**Affiliations:** Department of Reproductive Health and Research, World Health Organization, UNDP/UNFPA/UNICEF/WHO/World Bank Special Programme of Research, Development and Research Training in Human Reproduction (HRP), Avenue Appia 20, Geneva, 1201 Switzerland; Medical Statistics deparment, London School of Hygiene and Tropical Medicine, London, UK; Department of Obstetrics and Gynecology, Women’s Health Hospital, Faculty of Medicine, Assiut University, Assiut, Egypt; Centro Rosarino de Estudios Perinatales, Rosario, Argentina; Department of Obstetrics and Gynaecology, Yong Loo Lin School of Medicine, National University Hospital Singapore, Singapore, Singapore; Birmingham Women’s NHS Foundation Trust, Birmingham, UK; Department of Obstetrics and Gynaecology, College of Medicine, University of Ibadan, Ibadan, Nigeria; Department of Medical Education, K L E Society’s J N Medical College, Belgaum, India; Effective Research Care Unit, University of Witwatersrand, University of Fort Hare, East London, South Africa; Department of Obstetrics and Gynaecology, Khon Kaen University, Khon Kaen, Thailand; Department of Obstetrics and Gynecology, School of Medicine, Makerere University College of Health Sciences, Kampala, Uganda; Department of Obstetrics and Gynaecology, Kenyatta National Hospital, University of Nairobi, Nairobi, Kenya; Department of Social Medicine, Ribeirão Preto Medical School, University of São Paulo, Ribeirão Preto, SP Brazil

**Keywords:** Postpartum haemorrhage, Room temperature stable carbetocin, Oxytocin, Non-inferiority trial

## Abstract

**Background:**

Postpartum haemorrhage (PPH) is the leading cause of maternal mortality in low-income countries and contributes to nearly a quarter of maternal deaths globally. The current available interventions for prevention of postpartum haemorrhage, oxytocin and carbetocin, are limited by their need for refrigeration to maintain potency, as the ability to maintain a cold chain across the drug distribution and storage network is inconsistent, thus restricting their use in countries with the highest burden of maternal mortality. We describe a randomized, double-blind non-inferiority trial comparing a newly developed room temperature stable formulation of carbetocin to the standard intervention (oxytocin) for the prevention of PPH after vaginal birth.

**Methods/design:**

Approximately 30,000 women delivering vaginally will be recruited across 22 centres in 10 countries. The primary objectives are to evaluate the non-inferiority of room temperature stable carbetocin (100 μg intramuscular) versus oxytocin (10 IU intramuscular) in the prevention of PPH and severe PPH after vaginal birth. The primary endpoints are blood loss ≥500 mL or the use of additional uterotonics (composite endpoint required by drug regulatory authorities) and blood loss ≥1,000 mL (WHO requirement). Non-inferiority will be assessed using a two-sided 95 % confidence interval for the relative risk of the above endpoints for room temperature stable carbetocin versus oxytocin. The upper limit of the two-sided 95 % confidence interval for the relative risk for the composite endpoint of blood loss ≥500 mL or the use of additional uterotonics, and for the endpoint of blood loss ≥1,000 mL, will be compared to a non-inferiority margin of 1.16 and 1.23, respectively. If the upper limit is below the corresponding margin, non-inferiority will have been demonstrated. The safety analysis will include all women receiving treatment. Safety and tolerability will be assessed by a review of adverse events, by conducting inferential testing with significance levels for between-group comparisons.

**Discussion:**

If the results of the study show that room temperature stable carbetocin is a safe and effective alternative to oxytocin, this could have a substantial impact on the prevention of postpartum haemorrhage and maternal survival worldwide.

**Trial registration:**

ACTRN12614000870651 (14 August 2014)

**Electronic supplementary material:**

The online version of this article (doi:10.1186/s13063-016-1271-y) contains supplementary material, which is available to authorized users.

## Background

Postpartum haemorrhage (PPH) is defined as a blood loss of ≥500 mL within 24 hours of delivery, while severe PPH (sPPH) is defined as a blood loss of ≥1,000 mL within the same time frame [1]. PPH is the leading cause of maternal mortality in low-income countries, contributing to nearly a quarter of maternal deaths globally. In addition, PPH is a significant contributor to severe maternal morbidity and long-term disability, as well as to a number of other severe maternal conditions generally associated with more substantial blood loss, including shock and organ dysfunction [2–4]. Improving health care for women during childbirth in order to prevent and treat PPH is an essential step towards the achievement of the United Nations Millennium Development Goals.

The majority of deaths due to PPH could be avoided through the use of prophylactic uterotonics during the third stage of labour and by timely and appropriate management. Injectable oxytocin has been recommended by the World Health Organization (WHO) for routine use during the third stage of labour and is the preferred drug for the prevention and management of blood loss after childbirth; however, several studies have demonstrated that oxytocin loses potency in field conditions, particularly in tropical climates [1, 5, 6]. To decrease potency loss due to degradation, oxytocin must be either stored at controlled room temperature (25 °C or lower) for a restricted amount of time or refrigerated (2 °C to 8 °C), making its use difficult in low resource settings. To ease this barrier, several groups have been researching heat stable oxytocin formulations [5]. Though some progress has been made in this area, there is currently no heat stable oxytocin formulation for therapeutic use [7].

Carbetocin (1-deamino-1-monocarba-(2-O-methyltyrosine)-oxytocin) is a long-acting synthetic agonist analogue of the human oxytocin, and it appears to be a promising agent in the prevention of PPH following vaginal delivery. The clinical and pharmacological properties of carbetocin are similar to those of oxytocin. However, carbetocin is a more stable molecule and induces a prolonged uterine response, when administered postpartum, in terms of both amplitude and frequency of contractions due to its longer half-life [8–10]. In a Cochrane systematic review including 11 studies (2,635 women), the use of carbetocin resulted in a statistically significant reduction in the use of additional/therapeutic uterotonics compared to oxytocin for those who underwent caesarean delivery (risk ratio (RR) 0.62; 95 % confidence interval (CI) 0.44 to 0.88; four trials, 1,173 women), but not for vaginal delivery. Compared to oxytocin, carbetocin was associated with reduced uterine massage following both caesarean delivery (RR 0.54; 95 % CI 0.37 to 0.79; two trials, 739 women) and vaginal delivery (RR 0.70; 95 % CI 0.51 to 0.94; one trial, 160 women). There were no statistically significant differences between carbetocin and oxytocin in terms of risk of any PPH or in risk of sPPH [11].

Carbetocin is currently approved in multiple countries for the prevention of PPH during caesarean section. The drug is licensed to be administered by slow intravenous (IV) single injection at a dose of 100 μg. Its current formulation requires refrigeration.

A room temperature stable (RTS) variant of carbetocin has recently been developed and is now approved in the European Union; this variant differs from the current carbetocin formulation only in its excipients (Table 1). The stability data from long-term studies performed at 30 °C and 75 % relative humidity, as well as accelerated at 40 °C and 75 % relative humidity, indicate a shelf life of at least 36 months at 30 °C for the new RTS formulation of carbetocin. Carbetocin RTS therefore represents a promising intervention for reducing PPH, particularly in settings where cold storage is difficult to achieve and maintain. Here, we describe a planned randomized trial to evaluate the non-inferiority of carbetocin RTS versus oxytocin after vaginal delivery in the prevention of PPH and sPPH.

**Table 1:** Composition of carbetocin RTS and carbetocin

New carbetocin RTS formulation			Current carbetocin refrigerated formulation (Duratocin®, Ferring)^a^		
Component	Amount (mg/mL)	Function	Component	Amount (mg/mL	Function
Carbetocin	0.100	Active substance	Carbetocin	0.100	Active substance
Succinic acid	1.19	Buffer	Sodium chloride	9	Isotonicity agent
Mannitol	47.0	Isotonicity agent	Glacial acetic acid	to pH 3.8	pH adjustment
L-methionine	1.00	Antioxidant	Water for injection	1.0 mL	Solvent
Sodium hydroxide 2 N	to pH 5.45	pH adjustment	--	--	--
Water for injection	1.0 mL	Solvent	--	--	--

^a^Carbetocin 100 μg/mL was first approved in Canada in June 1997 and is currently registered in more than 70 countries under the trade names PABAL/DURATOCIN/LONACTENE/DURATOBAL

*RTS* room temperature stable

## Methods/design

### Primary objectives and hypotheses

The trial has two primary objectives: 1) To evaluate non-inferiority of carbetocin RTS versus oxytocin after vaginal delivery in the prevention of the composite endpoint “blood loss of 500 mL or more or the use of additional uterotonics” at one hour and up to two hours for women who continue to bleed after one hour; and 2) to evaluate non-inferiority of carbetocin RTS versus oxytocin in the prevention of sPPH (≥1,000 mL blood loss) at one hour and up to two hours for women who continue to bleed after one hour.

For both objectives the hypotheses are: 1) carbetocin RTS is non-inferior to oxytocin in terms of the proportion of women with blood loss ≥500 mL or the use of additional uterotonic drugs after vaginal delivery within a non-inferiority margin of 1.16 on the relative risk scale, and the proportion of women with blood loss ≥1,000 mL after vaginal delivery within a non-inferiority margin of 1.23 on the relative risk scale; and 2) carbetocin RTS is superior to oxytocin in terms of the proportion of women with blood loss ≥500 mL or use of additional uterotonic drugs, and in terms of the proportion of women with blood loss ≥1,000 mL after vaginal delivery. For each of the two primary endpoints, superiority will be tested if non-inferiority has been demonstrated.

Investigators and other assessors will be blinded to individual treatment allocation. The study will be conducted by the HRP/WHO, and WHO staff will also remain blinded to individual participant treatment allocation during the conduct of the trial.

### Study drug

Carbetocin RTS (Ferring, St. Prex, Switzerland) will be administered as a single IM dose of 100 μg in a 1 mL solution. Oxytocin 10 IU/mL (Syntocinon®, Novartis, Basel, Switzerland) will be administered as a single intramuscular (IM) dose in a 1 mL solution. In order to maintain the blindness of the trial, carbetocin RTS and oxytocin will be provided in 1 mL ampoules in consecutively numbered treatment packs arranged in dispensers and will be stored in a refrigerator at 2 °C to 8 °C.

### Study design

This clinical trial will be conducted in compliance with the clinical trial protocol, good clinical practice and the applicable regulatory requirements. The trial is planned to be conducted at 22 centres in 10 countries: Argentina, Egypt, India, Kenya, Nigeria, Singapore, South Africa, Thailand, Uganda and the United Kingdom. Approximately 30,000 women will be included in the trial. The recruitment period will be approximately 12 months in each country.

The care provider in charge of antenatal care visits at the hospital will inform potentially eligible pregnant women (women with no allergies to carbetocin, other oxytocin homologues or excipients, or serious cardiovascular disorders) about the trial.

At admission for labour at the hospital, the woman will be approached for participation in the trial when she is in early labour and if her vital signs are normal, and she is not stressed. The investigator will invite her to sign the informed consent. Informed consent will be obtained before any trial-related procedures are carried out.

During the second stage of labour when vaginal delivery is imminent, the woman will be randomized to receive either a single dose of oxytocin 10 IU IM or a single dose of carbetocin RTS 100 μg IM.

The investigator will administer the investigational medicinal product (IMP) immediately after the birth of the baby (preferably within one minute). Once the IMP has been administered, the investigator will follow the management of the third stage of labour as recommended in WHO guidelines and as detailed in the trial’s manual of operations.

Once the cord is clamped (1–3 minutes after delivery of the baby) and cut, the investigator will place a calibrated drape under the woman’s buttocks and blood loss will be measured for one hour or two hours postpartum if the bleeding continues beyond one hour (blood loss measuring details are provided in the trial’s manual of operations).

The woman will end her participation in the trial after her discharge or if she is transferred to a higher care unit, such as an intensive care unit (ICU). Data will be entered into a validated online database system compliant with the FDA 21 CFR part 11.

The trial master protocol was approved by the World Health Organization Research Ethics Review Committee. All sites received ethics approval from their institutional/national boards (see additional file 1 for the list of ethics committees that reviewed and approved the proposal).

### Inclusion/exclusion criteria

Women will be eligible for inclusion into this trial if they are expected to deliver vaginally, have a cervical dilatation equal to or less than 6 cm, have a known singleton pregnancy and have provide written informed consent. Women will not be eligible for study participation if they: 1) are in an advanced first stage of labour (>6 cm cervical dilatation) or are too distressed to understand, confirm and give informed consent regardless of cervical dilatation; 2) are non-emancipated minors (as per local regulations) without a guardian; 3) have been scheduled for a planned caesarean section; 4) have a birth considered to be an abortion according to local guidelines; 5) have known allergies to carbetocin, other oxytocin homologues or excipients in the medicinal products used in the trial; 6) have serious cardiovascular disorders; 7) have serious hepatic or renal disease; 8) have epilepsy; or 9) are not capable of giving consent due to other health problems such as obstetric emergencies (for example, antepartum haemorrhage) or mental disorder.

Participants will be free to discontinue the trial at any time without giving their reason(s). Participants who withdraw will not be replaced; that is, randomized numbers will be uniquely linked to each participant. Data collected up to that point will be included unless participants request the opposite. After a participant has completed the trial or has withdrawn early, usual treatment will be administered, if required, in accordance with the trial centre’s standard of care and generally accepted medical practice and depending on the participant’s individual medical needs.

### Randomization

The random allocation sequence will be generated centrally at WHO headquarters using computer-generated random numbers. Randomization will be stratified by country and will use permuted blocks, with an allocation ratio of 1:1. Participants will randomly be assigned within each country to one of the two treatment groups: oxytocin 10 IU IM or carbetocin RTS 100 μg IM. Allocation of the randomly generated sequence will be performed by consecutively numbered treatment packs arranged in dispensers.

### Primary and secondary endpoints

The primary and secondary endpoints are listed in Table 2. The trial is being conducted as an effectiveness trial with the objective of carbetocin RTS being registered for the indication “prevention of postpartum haemorrhage” by drug regulatory authorities if it is shown to be non-inferior or superior to oxytocin. The primary composite endpoint is blood loss ≥500 mL or use of additional uterotonics. For the purpose of evaluating clinical effectiveness and for the potential inclusion of carbetocin RTS in future WHO guidelines and the Model List of Essential Medicines, the more substantive endpoint of blood loss ≥1,000 mL is also a primary endpoint. Since the two primary endpoints serve independent objectives, there will be no adjustment for the type I error rate for multiplicity of endpoints.

**Table 2:** Primary and secondary endpoints

Primary endpoints
The proportion of women: • with blood loss of 500 mL or more or the use of additional uterotonics at one hour and up to two hours for women who continue to bleed after one hour; • with blood loss of 1,000 mL or more at one hour and up to two hours for women who continue to bleed after one hour.
Secondary endpoints
The proportion of women: • with blood loss of 500 mL or more at one hour (or two hours postpartum if the bleeding continues beyond one hour); • receiving additional uterotonics at one hour (or two hours postpartum if the bleeding continues beyond one hour); • receiving additional uterotonics up to time of discharge; • receiving blood transfusion up to time of discharge; • with manual removal of placenta up to time of discharge; • having additional surgical procedures (for example, suturing of cervix/high vaginal tear, exploration of uterine cavity under general anaesthetic, uterine compression suture, uterine or hypogastric ligation, hysterectomy) up to time of discharge; • with maternal death; • with composite outcome of maternal death or severe morbidity (admission to intensive care unit, hysterectomy, blood loss of two litres or more, uterine inversion) up to time of discharge Blood loss in mL at one hour (or two hours postpartum if the bleeding continues beyond one hour). The incidence and severity of adverse or serious adverse events up to the time of discharge. Newborn outcomes (vital status, Apgar score at 5 minutes, resuscitation of the baby, mechanical ventilation).

### Assessment of efficacy

Efficacy will be assessed using blood loss volume measurement per the two primary endpoints listed in Table 2. Blood loss will be measured using a calibrated drape [12], which will be placed under the participant’s buttocks once the cord is clamped (1–3 minutes after delivery of the baby) and cut. Blood loss will be measured for one hour, or for two hours postpartum, if the bleeding continues beyond one hour. Blood loss weight in grams will be converted to millilitres by dividing the figure in grams by 1.06 (blood density in grams per millilitre) [13].

### Assessment of safety

Comprehensive assessment of the safety of the study drugs will be performed throughout the course of the trial, from the time of the participant’s signature of informed consent until discharge. Trial centre personnel will report any adverse event (AE), whether observed by the investigator or reported by the participant.

The intensity of an AE will be classified using a three-point scale: mild ─ awareness of signs or symptoms, but no disruption of usual activity; moderate ─ event sufficient to affect usual activity; and severe ─ inability to work or perform usual activities. AEs will also be classified as possibly related to study drug (that is, there is evidence or argument to suggest a causal relationship between the AE and the study drug), or not related to study drug (that is, there is no reasonable evidence or argument to suggest a causal relationship between the AE and the study drug).

The outcome of an AE will be classified as: recovered/resolved (fully recovered or the condition has returned to the level observed at initiation of trial treatment); recovered/resolved with sequelae (resulted in persistent or significant disability/incapacity); recovering/resolving; not recovered/not resolved; or fatal.

An AE will be considered serious if it results in death, is life-threatening, requires in-patient hospitalization or prolongation of existing hospitalization, results in persistent or significant disability/incapacity, is a congenital anomaly/birth defect, or is an important medical event. If an investigator becomes aware of a serious AE after the participant’s hospital discharge, and he/she assesses the serious AE to have a reasonable possible causality to the study drug, the case will be reported, regardless of how long after the end of the trial the AE took place.

Since the amount of blood lost is collected as an efficacy endpoint, only the following blood loss events that fulfil the criteria for a serious AE will be recorded: ICU admission (adult ICU admission, not maternity/labour ward high care); surgical intervention for control of haemorrhage (hypogastric artery ligation or uterine compression sutures); peripartum hysterectomy, blood loss ≥ 2,000 mL; and uterine inversion.

### Sample size calculation

The two endpoints that guided the sample size estimation for the trial were sPPH (blood loss ≥1,000 mL), PPH (blood loss ≥500 mL) or the use of additional uterotonics. Severe PPH is a more serious endpoint that is closely associated to severe maternal morbidity and related to additional interventions. The sPPH endpoint is also less frequent and thus guided the overall sample size estimation.

**Fig. 1:**
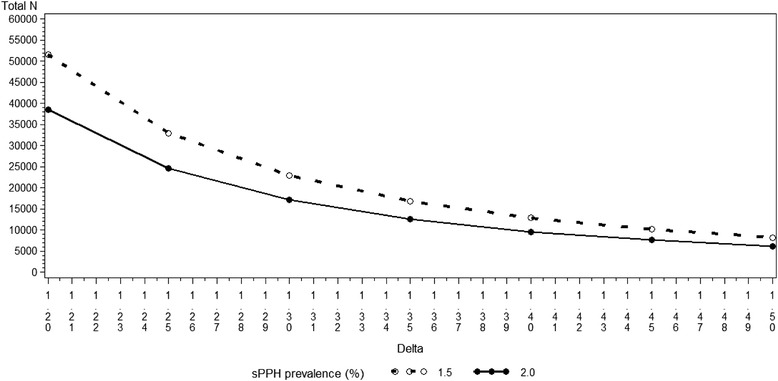
Total sample size for non-inferiority with equal proportions = 1.5 % or 2 %, 80 % power and different values of the margin on the relative scale

A non-inferiority design was chosen since the aim of the trial is to determine if carbetocin RTS is non-inferior in efficacy to the standard intervention (oxytocin 10 IU IM). In order to demonstrate non-inferiority within a margin of 0.46 %, with a power of 80 % and with a significance level of 2.5 %, a total of 29,082 women are needed assuming an equal sPPH prevalence of 2 % with both treatments, calculated by the standard formula (see Additional file 2 for the rationale of the choice of the margin) [14]. The margin of 0.46 % on the difference scale corresponds to 1.23 on the relative scale for the assumed prevalence. Figure 1 shows the total sample size required to assess non-inferiority at the 2.5 % level of significance (usually required for non-inferiority trials) for different scenarios. We also assumed, from a similar large trial [15], a 3 % loss of study participants who are eligible to contribute to the analysis due to exclusion of women with a caesarean section or abortion after randomization, and those who are not protocol-compliant (excluded only in the per-protocol analysis), bringing the sample size to 30,000. This sample size will provide 90 % power for a conventional two-sided 5 % test of superiority to detect a minimum significant difference between 1.5 % and 2 % in the rate of sPPH for the two treatment groups.

Similarly, we calculated the sample size for the composite endpoint defined as blood loss ≥500 mL or administration of additional uterotonics. With active management, the risk of this event is estimated as 16 % (personal communication) [15]. Assuming equal prevalence for this event of 16 % with both treatments, in order to demonstrate non-inferiority within a margin of 1.16 on the relative scale, with a power of 80 % and with a significance level of 2.5 %, a total of 6,242 women are needed. With 30,000 women the power obtained would be more than 99 % [14]. This sample size will provide a power of 99.5 % for a conventional two-sided 5 % test of superiority to detect a minimum significant difference between 16 % and 18 % in the occurrence of PPH or administration of additional uterotonics for the two treatment groups (Stata version 13.1).

### Analysis populations

Analyses will be performed in three populations: intention-to-treat (ITT), modified ITT, and per-protocol. The ITT population includes all randomized participants, using the treatment as randomized. The modified ITT analysis excludes women having a caesarean section after randomization and those withdrawing consent. The per-protocol population includes all participants randomized into the trial, who were compliant with, and treated according to, the protocol, and who fulfil the following criteria: compliance with all entry criteria, absence of major clinical trial protocol violations with respect to factors likely to affect the efficacy of treatment, and adequate compliance with trial medication (giving oxytocin or carbetocin to women as randomized). Declaring carbetocin RTS non-inferior to oxytocin will require non-inferiority to be demonstrated for both per-protocol and the modified ITT analyses. For testing the superiority of each endpoint, the modified ITT analysis will be the main analysis. The primary population for safety analysis will consist of all women receiving treatment.

### Statistics

Safety and tolerability will be assessed by a review of AEs, by conducting inferential testing with significance levels for between-group comparisons. The between-treatment difference for the occurrence of AEs will be tested using the stratified Mantel-Haenszel chi-square if frequencies are sufficiently high, or otherwise with exact methods taking stratification into account.

For efficacy, non-inferiority will be assessed using a two-sided 95 % confidence interval (CI) for the relative risk (RR) of sPPH and of PPH or additional uterotonic use (carbetocin RTS versus oxytocin). The upper limit of the two-sided 95 % CI for the RR for the composite endpoint of blood loss ≥500 mL or additional uterotonic use will be compared to the non-inferiority margin of 1.16. The upper limit of the two-sided 95 % CI for the RR for the endpoint of blood loss ≥1,000 mL will be compared to the non-inferiority margin of 1.23. If the upper limit is below the corresponding margin, non-inferiority will have been demonstrated. This upper limit is the same as the upper limit of the one-sided 97.5 % CI; therefore, the significance level for the non-inferiority test will be 2.5 % (one-sided). For each endpoint, to declare carbetocin RTS non-inferior to oxytocin, we will require non-inferiority to be demonstrated for both PP and the modified ITT analyses. If non-inferiority is demonstrated, a two-tailed superiority test will be conducted at 5 % level of significance. Interpretation will be as in Fig. 1 of Piaggio et al. [16]. Comparisons will also be expressed as risk differences with 95 % CIs.

The statistical technique used to conduct tests and obtain CIs for the main endpoints will be a logistic model with a binary endpoint, a binomial distribution and the log link to obtain RRs. The identity link will be used to obtain risk differences. Stratifying variables (country) will be included in the model. The model will be fitted using SAS Software version 9.3 (SAS Institute Inc., Cary, NC, USA). A separate model will be fitted for each of the two primary endpoints.

We will use the RR as measure of treatment effect on the relative scale for the two primary endpoints. For the composite endpoint of blood loss ≥500 mL or use of additional uterotonics, the margin has been justified as 1.2 on the odds ratio (OR) scale and 1.16 on the RR scale. Heterogeneity across centres will be assessed by using a term in the logistic model for the interaction between treatment and centres. If there is heterogeneity between the centres for any of the results, the possible causes will be explored.

The secondary binary endpoints of blood loss ≥500 mL, use of additional uterotonics, blood transfusion, manual removal of placenta, additional surgical procedures, maternal death and the composite endpoint of maternal death or severe morbidity, will be analysed using the modified ITT population and will be assessed only for conventional superiority using risk differences and relative risks with 95 % CIs estimated with the same techniques described for the main endpoints.

The secondary outcome of blood loss in millilitres will be analysed using the log transformation. This is based on the following: 1) the blood loss distribution is positively skewed; 2) different distributions were fitted to blood loss data from a large trial [15] and the lognormal distribution was found to have a very good fit (personal communication); and 3) the lognormal distribution was used for blood loss data in the literature [13]. Therefore, a lognormal distribution will be fitted to the blood loss data and the probabilities of blood loss ≥500 mL and of ≥1,000 mL will be compared between treatments using parametric methods. The quantiles will be compared between treatments using quantile regression [17].

### Trial oversight

The trial will be overseen by two committees. The Trial Steering Committee (TSC) is composed of the WHO/HRP coordination team, investigators from the 10 participating countries and five independent members with expertise in clinical, statistical and scientific disciplines. The TSC should provide oversight of the progress of the trial and ensure the trial is conducted in accordance with the principles of good clinical practice (GCP).

The Data and Safety Monitoring Committee (DSMC), with no direct involvement in the trial, will handle any ethical issues that may arise while the trial is in progress and will scrutinize two interim analyses, the first to look at safety when 5,000 participants have been recruited, and the second conducted when 15,000 women have been recruited to look at both safety and efficacy. The interim analyses will be masked to trial investigators, trial statistician, WHO, Merck for Mothers and Ferring staff, but not to DSMC members. The DSMC will be expected to provide an ongoing risk-benefit evaluation that addresses the uncertainty necessary to continue.

## Discussion

PPH is one of the major contributors to maternal mortality and morbidity worldwide. Prevention of PPH is therefore of great importance in the pursuit of improved health care for women. The decision to proceed with a large, randomized controlled trial to address this unmet medical need by evaluating the effectiveness of carbetocin RTS compared to oxytocin was based on several considerations. This included the frequent concerns about the quality of oxytocin and its stability in some developing countries, the potential advantage of carbetocin RTS due to its longer half-life (than that of oxytocin), especially when administered intramuscularly, and its heat stability. This study will be conducted under conditions where both study drugs, oxytocin and carbetocin RTS, will be kept under strict refrigeration. If the trial meets its primary endpoint of non-inferiority to oxytocin, the carbetocin RTS formulation will be made available in high-burden countries at an accessible public sector price. This would have major implications for expanding access to effective care and could have a substantial impact on maternal survival.

The risk-benefit relationship was carefully considered in the planning of the trial, as was the selection of the dose of carbetocin RTS. The selected dose for the carbetocin RTS IM injection is 100 μg, the same as the dose approved for IV injection following caesarean section. The safety profile of a 100 μg dose of the carbetocin refrigerated formulation, when administered intravenously, is derived from clinical studies as well as from pharmacovigilance activities that comprise nearly 5 million women exposed. Reported AEs during clinical trials when administered by IV after caesarean section under spinal or epidural anaesthesia are headache, tremor, hypotension, flushing, nausea, abdominal pain, pruritus, anaemia, dizziness, chest pain, dyspnoea, metallic taste and vomiting. The AEs observed were of the same type and frequency as those observed with the comparator, oxytocin. In addition, efficacy and safety data on 100 μg of the carbetocin refrigerated formulation delivered intramuscularly during vaginal delivery is available from several published randomized clinical trials [11, 18, 19]. Although the population from whom these safety data are generated is different from the study population of this protocol, women undergoing caesarean section are expected to be at the same or higher risk of AEs than women having a vaginal delivery. It is therefore expected that a carbetocin RTS dose of 100 μg IM, when used in women after vaginal delivery, will be efficacious and have a safety profile that is equal to or better than the well-characterized safety profile of 100 μg IV when used in women following caesarean section.

The study has several strengths. It is a global, multi-centred, randomized, double-blind, active comparator controlled study. The active comparator is oxytocin, which is the most commonly recommended drug for PPH prophylaxis and considered the current gold standard in WHO guidelines. The 10 IU dose of oxytocin was chosen as this is currently the most commonly used dose in clinical practice internationally when IM administration is practiced. Blood loss of ≥1,000 mL is a primary endpoint in this trial, as it was considered as one of the three critical endpoints (together with blood transfusion and maternal death) for the earlier 2007 WHO recommendations for PPH prevention where outcomes were rated by an independent panel [1]. We also used reliable estimates of prevalence of blood loss ≥1,000 mL under active management with oxytocin from different trials and systematic reviews. Additionally, an external DSMC has been established for the ongoing assessment of the risk-benefit ratio. One limitation of our study is that the predicted sample size makes assumptions that in reality might not hold. For example, if the efficacy of oxytocin is much better than that of carbetocin RTS, this would imply that non-inferiority is not likely to be demonstrated and the trial should be stopped for futility. If oxytocin is just slightly better than carbetocin RTS, the predicted sample size could have been underestimated, but this is not likely given previous evidence [11].

Results of this trial will be extremely useful, particularly in tropical settings, where cold storage is difficult to achieve and maintain. Should this trial demonstrate that carbetocin RTS is non-inferior to oxytocin in preventing postpartum haemorrhage, in settings where the cold chain cannot be guaranteed, oxytocin could be replaced by carbetocin RTS as the uterotonic used during the third stage of labour. In addition, and based on the tripartite agreement signed between WHO/HRP, Merck for Mothers and Ferring Pharmaceuticals, carbetocin RTS could be available in high-burden countries at a public sector price comparable to oxytocin’s price. This could have major implications for preventing PPH and a substantial impact on maternal survival worldwide.

### Trial status

The trial has started recruitment in five countries. The other five countries are expected to receive regulatory authorities’ approval before April 2016.

## Additional files

Additional file 1: Countries ethics committees list and status of the project approval request. (DOCX 15 kb) Additional file 2: Rationale for the choice of the non-inferiority trial the choice of the non-inferiority trial [20-22]. (DOCX 24 kb)

## Abbreviations

AE: adverse event
CI: confidence interval
DSMC: Data Safety Monitoring Committee
GCP: good clinical practice
HRP: the UNDP/UNFPA/UNICEF/WHO/World Bank Special Programme of Research, Development and Research Training in Human Reproduction
IM: intramuscular
IMP: investigational medicinal product
ITT: intention to treat
IU: international unit
IV: intravenous
mL: millilitres
PPH: postpartum haemorrhage
RHR: reproductive health and research
RR: risk ratio
RTS: room temperature stable
SAE: serious adverse event
sPPH: severe postpartum haemorrhage
TSC: Trial Steering Committee
WHO: World Health Organization
