# Supplementary material for: Room temperature stable carbetocin for the prevention of postpartum haemorrhage during the third stage of labour in women delivering vaginally: study protocol for a randomized controlled trial
Source: Trials. 2016 Mar 17;17:143. doi: 10.1186/s13063-016-1271-y (PMC4794812; doi:10.1186/s13063-016-1271-y)
Supplement: Additional file 1: — Countries ethics committees list and status of the project approval request. (DOCX 15 kb) [file 13063_2016_1271_MOESM1_ESM.docx]

Countries ethics committees list and status of the project approval request.

| Site Country | Reg./Ethics Type | Ethics Name | Status |
| --- | --- | --- | --- |
| Argentina | Central Ethics Committee | Comité de Ética Independiente del Centro Rosarino de Estudios Perinatales | Approved |
| Argentina | Central IRB | Comité de Docencia e Investigación Hospital Roque Saenz Peña | Approved |
| Argentina | Central IRB | Comité de Docencia e Investigación Maternidad Martin | Approved |
| Egypt | Local Ethics Committee | Assiut university, Faculty of Medicine, Medical Ethics Committee | Approved |
| India | Local Ethics | Ethics Committee of the KLE University | Approved |
| India | Local Ethics | Institutional Ethics Committee, S Nijalingappa Medical College and HSK Hospital & RC | Approved |
| India | Local Ethics | Institutional Ethics Committee, Shri B.M. Patil Medical College, Hospital and Research Centre | Approved |
| Kenya | Local Ethics Committee | KEMRI Ethics Review Committee | Approved |
| Nigeria | Local Ethics Committee | University College Hospital Health Research Ethics Committee | Approved |
| Nigeria | Local Ethics | University College Hospital Health Research Ethics Committee | Approved |
| Singapore | Central IRB | National Healthcare Group Domain Specific Review Board | Approved |
| South Africa | Local Ethics Committee | Wits Health Consortium | Approved |
| South Africa | Local Ethics | Wits Health Consortium | Approved |
| Thailand | Local Ethics | The Khon Kaen University Ethics Committee for Human Research | Approved |
| Uganda | Central Ethics Committee | Uganda National Council of Science and Technology (UNCST) | Approved |
| Uganda | Local Ethics Committee | Makerere University School of Biomedical Sciences | Approved |
| United Kingdom | Central Ethics Committee | NRES Committee East Midlands - Northampton | Approved |
| United Kingdom | Local Ethics | R&D - University Hospitals Birmingham | Approved |

Green row : approval granted

White row : approval pending
