# Supplementary material for: Room temperature stable carbetocin for the prevention of postpartum haemorrhage during the third stage of labour in women delivering vaginally: study protocol for a randomized controlled trial
Source: Trials. 2016 Mar 17;17:143. doi: 10.1186/s13063-016-1271-y (PMC4794812; doi:10.1186/s13063-016-1271-y)
Supplement: Additional file 2: — Rationale for the choice of the non-inferiority trial the choice of the non-inferiority trial [20-22]. (DOCX 24 kb) [file 13063_2016_1271_MOESM2_ESM.docx]

**Additional file 2 – Rationale for the choice of the non-inferiority trial**

In a non-inferiority trial, we need evidence that the comparator is effective compared to placebo, because if it is not, the non-inferiority trial might demonstrate non-inferiority of two ineffective treatments. The ‘comparator’ treatment is Oxytocin 10 IU/mL with management of the third stage of labour as recommended in WHO guidelines, described under *Study Design*. Placebo is expectant management, in which signs of placental separation are awaited and the placenta is delivered spontaneously. Since we did not find trials in which the comparator was compared to placebo using these definitions, we decided to use reliable estimates of prevalence of blood loss ≥1000 mL under active management with oxytocin as part of the package and under expectant management, coming from different trials or systematic reviews. With active management, the risk of sPPH was estimated as 2% in a multicenter large trial (15). With expectant management, the systematic review of Carroli et al (20) reported a prevalence of 3.84%. We considered that the prevalence estimate of 2% for active management with oxytocin as part of the package, and of 3.84% for expectant management were reliable, so that the effect of the ‘comparator’ would be 1.84%.

To preserve 75% of the benefit of oxytocin over expectant management, assuming an effect of the ‘comparator’ of 1.84%, we need a margin of non-inferiority of Δ = (1-0.75) x 1.84=0.46%. In relative terms (relative risk), this gives a margin of non-inferiority of [2 + 0.46] / 2 = 2.46 / 2) = 1.23.

The above margin Δ reflects the clinical judgment about how much of the effect of the active control should be preserved by ruling out a loss of Δ (21, 22). Preserving a higher percentage (say 80% or 90%) will push the sample size calculations very high while a smaller percentage (say 70%) may not be considered acceptable. We considered rounding-up the margin to 0.5%, which would require about 25,000 subjects, but we wanted to be conservative and we decided to recruit 30,000 subjects.
